# *Trichinella* spp. in Wild Boars (*Sus scrofa*), Brown Bears (*Ursus arctos*), Eurasian Lynxes (*Lynx lynx*) and Badgers (*Meles meles*) in Estonia, 2007–2014

**DOI:** 10.3390/ani11010183

**Published:** 2021-01-14

**Authors:** Age Kärssin, Liidia Häkkinen, Annika Vilem, Pikka Jokelainen, Brian Lassen

**Affiliations:** 1 Estonian Veterinary and Food Laboratory, 51006 Tartu, Estonia; liidia.hakkinen@vetlab.ee (L.H.); annika.vilem@vetlab.ee (A.V.); 2 Institute of Veterinary Medicine and Animal Sciences, Estonian University of Life Sciences, 51006 Tartu, Estonia; PIJO@ssi.dk; 3 Laboratory of Parasitology, Department of Bacteria, Parasites and Fungi, Infectious Disease Preparedness, Statens Serum Institut, 2300 Copenhagen, Denmark; 4 Faculty of Veterinary Medicine, University of Helsinki, 00014 Helsinki, Finland; 5 Research Group for Global Capacity Building, Division for Global Surveillance, National Food Institute, Technical University of Denmark, 2800 Copenhagen, Denmark; brlas@food.dtu.dk

**Keywords:** foodborne, game meat, *Trichinella*, wildlife, zoonosis

## Abstract

**Simple Summary:**

Trichinellosis is an important foodborne zoonosis. In Estonia, *Trichinella* infections are endemic in wild animals. This paper summarizes findings of *Trichinella*-parasites during an 8-year period in Estonia in selected host species: wild boars, brown bears, Eurasian lynxes, and badgers. The results highlight that testing wildlife hunted for human consumption for *Trichinella* is important, and that there is room for improvement in the proportion of hunted animals tested.

**Abstract:**

In this study, we summarize *Trichinella* findings from four wild, free-ranging host species from Estonia during 2007–2014. *Trichinella* spp. larvae were detected in 281 (0.9%, 95% confidence interval (CI) 0.8–1.0) of 30,566 wild boars (*Sus scrofa*), 63 (14.7%, 95% CI 11.6–18.3) of 429 brown bears (*Ursus arctos*), 59 (65.56%, 95% CI 55.3–74.8) of 90 Eurasian lynxes (*Lynx lynx*), and three (60.0%, 95% CI 18.2–92.7) of five badgers (*Meles meles*). All four European *Trichinella* species were detected: *T. britovi* in 0.7% of the wild boars, 7.2% of the brown bears, 45.6% of the lynxes, and 40.0% of the badgers; *T. nativa* in 0.1% of the wild boars, 5.8% of the brown bears, and 20.0% of the lynxes; *T. pseudospiralis* in 0.02% the wild boars; and *T. spiralis* in 0.03% of the wild boars and 4.4% of the lynxes. The results include the first description from Estonia of *T. britovi* in brown bear and badgers, *T. pseudospiralis* in wild boars, and *T. spiralis* in wild boars and lynxes. The results indicate high infection pressure in the sylvatic cycles across the years—illustrating continuous risk of spillover to domestic cycles and of transmission to humans.

## 1. Introduction

*Trichinella* spp. are zoonotic parasitic nematodes that can be transmitted to humans by consumption of undercooked or raw meat of infected animals. A multicriteria-based approach placed *Trichinella spiralis* as the third and *Trichinella* spp. other than *T. spiralis* as the fifth on a prioritization ranking list of foodborne parasites in Europe, and the fourth and the third, respectively, in Eastern Europe [1].

Meat of game animals, especially meat of wild boars (*Sus scrofa*), is considered one of the main sources of *Trichinella* infections for humans in Europe [2], and it is acknowledged as the main source in Estonia [3]. Cases of human trichinellosis have been reported from Estonia [4], and the proportion testing positive for antibodies against *Trichinella* spp. was 3.1% in the general adult human population and 4.9% among hunters [5].

*Trichinella* spp. are endemic in wildlife in Estonia [6]. A high proportion, 42.1%, of investigated wild boars that were hunted in 2012–2013 tested positive for antibodies against *Trichinella* [7], and the biomass of *Trichinella* has increased in the main reservoir hosts raccoon dogs (*Nyctereutes procyonoides*) and red foxes (*Vulpes vulpes*) [6,8]. To add to the knowledge on epidemiology of these zoonotic parasites, the aim of this study was to summarize *Trichinella* findings during 2007–2014 in selected sylvatic hosts that are hunted in Estonia: wild boars, brown bears (*Ursus arctos*), Eurasian lynxes (*Lynx lynx*), and badgers (*Meles meles*).

## 2. Materials and Methods

### 2.1. Ethics

No animals were killed for the purpose of this study. No data of the hunters were handled in this study.

### 2.2. Setting

Estonia is a Baltic country located in northeastern Europe. Altogether 158,670 wild boars, 348 brown bears, 797 lynxes and 1527 badgers were hunted in Estonia in 2007–2014 [9]. Meat of all these host species included in this study is eaten in the country.

### 2.3. Samples and Data

The muscle samples included in this study were collected from wild boars, brown bears, lynxes, and badgers, primarily from the predilection muscle groups (diaphragm, muscles of foreleg, or tongue), from across Estonia in 2007–2014 by hunters and by meat inspectors in game meat processing plants. The samples were sent to the Estonian Veterinary and Food Laboratory for *Trichinella* testing as part of meat inspection, either for primary or confirmatory testing.

Data on sex and age category of the animal, the county where the animal was hunted, and the year when the animal was hunted were extracted from the submission forms that accompanied the samples. The age category of wild boars, brown bears and lynxes was ‘juvenile’ for animals the hunters estimated to be up to 2 years of age and ‘adult’ for animals the hunters estimated to be over two years of age. The counties were categorized into eastern vs. western counties and southern vs. northern counties (Table 1).

### 2.4. Artificial Digestion

The artificial digestion of the samples was carried out at the Estonian Veterinary and Food Laboratory, which is the national reference laboratory for parasites with its three regional laboratories. The laboratories are accredited by ISO 17025 and authorized as official laboratories for *Trichinella* digestion analyses according to EU 2075/2005 Annex I Chapter 1 [10]. One of the regional laboratories used Stomacher method according to EU 2075/2005 Annex I Chapter II [10] until 2009.

The testing included both primary and confirmatory testing. Other laboratories performing *Trichinella* testing send positive samples to the national reference laboratory for confirmation and species identification.

A minimum of 10 g muscle tissue was used from each animal in a pooled sample and 50 g for an individual sample, with the exception that in 2007, one regional laboratory used 5 g muscle tissue as the minimum in pooled samples for up to 20 animals. If a pooled sample was positive, the pool was divided into smaller pools and individual samples were tested to identify the infected animals.

If larvae were found, they were identified morphologically, counted, washed with tap water, and stored in ethanol, according to the procedures recommended by the European Union Reference Laboratory for Parasites [11]. The number of larvae per gram of muscle tissue (lpg) was calculated for each positive animal.

### 2.5. Molecular Analysis

Larvae collected in 2007–2010 were identified to the species level at the European Union Reference Laboratory [11], and larvae collected since 2011 at the Estonian Veterinary and Food Laboratory. The same multiplex PCR [12] was used for all the analyses. The method does not include sequencing.

### 2.6. Statistical Analyses

Only results from primary testing were used for prevalence estimates. Animals were excluded from statistical analyses if their individual infection status could not be determined, due to testing as part of a pooled sample followed by unsuccessful identification of the infected individuals.

We compared the prevalence estimates by sex, by age group, by eastern vs western counties and by southern vs northern counties, using two-by-two tables. In addition, we report univariable odds ratios for testing positive for *Trichinella*, using these same dichotomous variables, as well as counties as dummy variables and years as dummy variables. 

For the statistical analyses, we used Microsoft Excel, OpenEpi and R [13,14]. We report 95% confidence intervals (95% CI, Mid-P Exact) for proportions. Associations were considered statistically significant if two-tailed *p* < 0.05.

## 3. Results

The proportion of animals included in this study from the officially reported hunting bag of the study period was 19.3% for wild boars, 123.6% for brown bears, 12.0% for lynxes and 0.3% for badgers [9]. A total of 44 wild boars and two lynxes were excluded from statistical analyses, because their individual infection status could not be determined. The final sample was 31,090 animals tested as primary testing (Table S1), and 20 positive animals (15 wild boars, one brown bear and four lynxes) that had been tested for confirmatory purposes. Data on larval burden were missing for one wild boar and two lynxes. For *Trichinella* spp. species identification, altogether 426 larval samples were tested; *Trichinella* species was not determined in 70 (16.4%) of the larval samples.

**Figure 1:**
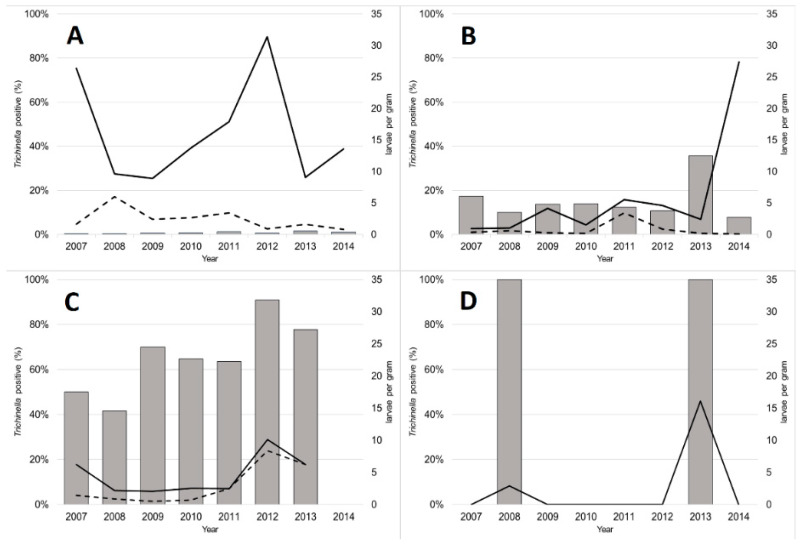
Percentage of *Trichinella* spp. positive animals; Mean (solid line) and median (dashed line) number of larvae per gram tissue in tested wild boars (*Sus scrofa*, **A**), brown bears (*Ursus arctos*, **B**), Eurasian lynxes (*Lynx lynx*, **C**) and badgers (*Meles meles*, **D**), 2007–2014, Estonia.

Of the altogether 31,090 animals tested as primary testing, 406 (1.3%, 95% CI 1.2–1.4) were positive for *Trichinella* spp. larvae. Altogether 281 (0.9%, 95% CI 0.8–1.0) of the 30,566 wild boars, 63 (14.7%, 95% CI 11.6–18.3) of the 429 brown bears, 59 (65.6%, 95% CI 55.3–74.8) of the 90 lynxes, and three (60.0%, 95% CI 18.2–92.7) of the five badgers were *Trichinella* positive (Table 1). In wild boars and lynxes, a higher prevalence was observed in adults than in juveniles (*p* = 0.003 and *p* = 0.045, respectively) (Table 1). In wild boars, the prevalence was higher in the western counties than in the eastern counties (*p* < 0.001), and in the northern counties than in the southern counties (*p* = 0.026) (Table 1). The prevalence in lynxes was higher in the eastern counties than in the western counties (*p* = 0.020), and in the northern counties than in the southern counties (*p* = 0.045). The prevalence varied by year from 0.4% to 1.6% in wild boars, from 7.9% to 36.7% in brown bears, from 41.7% to 90.9% in lynxes, and from 0.0% to 100.0% in badgers (Figure 1, Table 1). In wild boars, the prevalence was higher in 2011 (*p* = 0.001), 2013 (*p* < 0.001), and 2014 (*p* < 0.001) than it was in 2007 (Table 1).

The larval burden appeared generally higher in wild boars than in brown bears and lynxes (Figure 1, Table 1). Nine wild boars had more than 100 lpg. 

**Figure 2:**
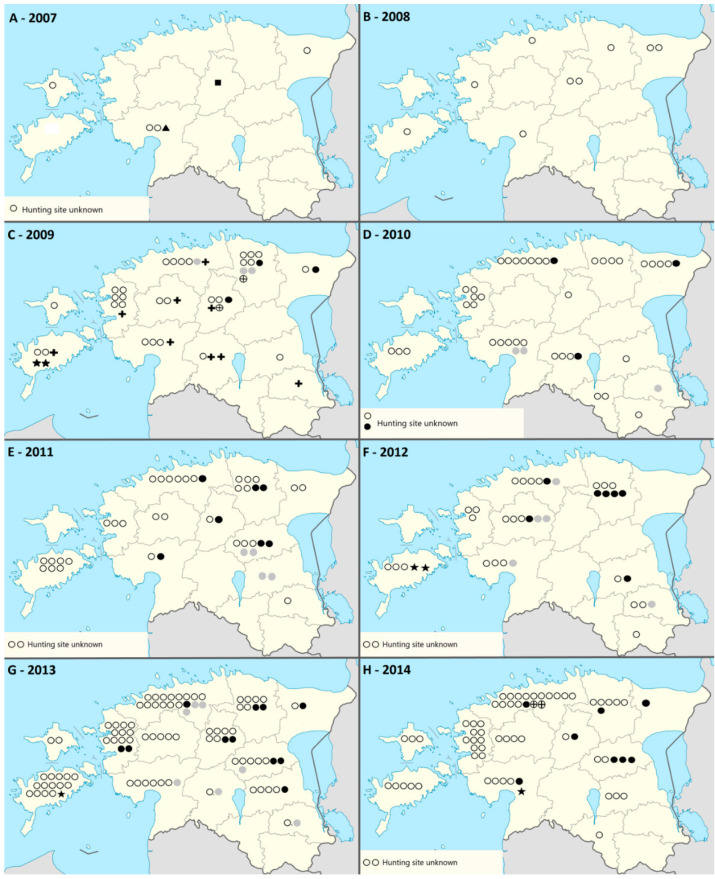
*Trichinella* species distribution in the tested animals that were positive and *Trichinella* species identification was successful, 2007–2014 (**A**–**H**, respectively), Estonia. *T. britovi*—ring, *T. nativa*—black dot, *T. pseudospiralis*—star; *T. spiralis*—cross, *T. britovi* and *T. nativa*—grey dot, *T. britovi* and *T. spiralis*—ring with cross.

Mono-species *Trichinella* infection was found in 97.5% (95% CI 95.0–99.0) of the wild boars, 94.3% (95% CI 85.4–98.5) of the brown bears, 69.4% (95% CI 55.5–81.0) of the lynxes, and all badgers that were positive and had the *Trichinella* species identified. The *Trichinella* species that were detected are shown by county and by year in Figure 2, Table 1 and Table S2. The isolates of 2007–2010 were deposited in International *Trichinella* Reference Centre [11].

*Trichinella britovi* was the most common *Trichinella* species found in all the investigated host species. It was found in animals from all counties (Figure 2, Table S2), in 0.7% (95% CI 0.6–0.8) of wild boars, 7.2 % (95% CI 5.1–10.0) of brown bears, 45.6% (95% CI 35.5–55.9) of lynxes, and 40.0% (95% CI 7.4–81.8) of badgers (Table 2). *Trichinella britovi* infections were found in 31 brown bears: in five hunted in Ida-Virumaa in 2007, 2010 and 2011; in four hunted in Harjumaa in 2010, 2011, 2012, and 2013; in four hunted in Järvamaa in 2008 and 2013; in four hunted in Jõgevamaa in 2011 and 2013; in two hunted in Läänemaa in 2009 and 2010; in four hunted in Lääne-Virumaa in 2009, 2010 and 2013; in one hunted in Põlvamaa in 2012; in two hunted in Pärnumaa in 2008 and 2012; and in three hunted in Tartumaa in 2009, 2013 and 2014; and in two badgers, which had been hunted in Lääne-Virumaa and Viljandimaa in 2013—these are the first confirmed findings of this parasite species in these host species in Estonia (this study; [15]).

The second most common *Trichinella* species was *T. nativa*, was found in all the investigated host species except badgers. Infected animals originated from 11 of the 15 counties; no findings were detected on the islands Hiiumaa and Saaremaa, and the southeastern counties Võrumaa and Valgamaa (Fig. 2, Table S2). *Trichinella nativa* was found in 0.1% (95% CI 0.0–0.1) of wild boars, 5.8 % (95% CI 3.9–8.4) of brown bears, and 20.0% (95% CI 12.7–29.2) of lynxes (Table 2).

*Trichinella pseudospiralis* was found in 2009 for the first time in wild boars in Estonia (Table 1; [11,16]). During the study period, this species was found in 0.02% of wild boars (Table 2); the prevalence was highest in Saaremaa, 0.2% (95% CI 0.1–0.5; Table S2).

The first *Trichinella spiralis* finding in a game animal in Estonia was identified in a lynx hunted in 2008 (shipped and tested in 2009), and further findings were detected in wild boars and lynxes hunted in 2009. The species was found in 0.03% (95% CI 0.0–0.05) of wild boars and 4.4% (95% CI 1.4–10.4) of lynxes (Table 2). It was detected in nine counties: Harjumaa, Järvamaa, Läänemaa, Lääne-Virumaa, Põlvamaa, Pärnumaa, Raplamaa, Saaremaa, and Viljandimaa (Table S2).

## 4. Discussion

The high number of observations in this study add substantially to the knowledge on epidemiology of *Trichinella* spp. in Estonia and highlight that wildlife, including game animals, have a key role in it. *Trichinella* spp. are important zoonotic parasites in the country and the region [17], and it is crucial that the One Health approaches addressing them cover not only domestic animals and humans, but also wildlife.

It should be emphasized that hunted animals are always a convenience sample: hunting periods affect the age of the animals included in the sample, and the representativeness of a hunter-harvested sample is challenging to evaluate. Moreover, it should be noted that e.g., animals injured in traffic accidents or hunted illegally are not included in the official hunting statistics. This could explain the higher number of brown bears in our sample than in the hunting bags.

The sampling was done by hunters and veterinary inspectors, who were advised to sample from the predilection muscle groups, if these were available [10]. The sampling was not supervised by the authors, and possible variation in sample material may have affected the results to the direction of underestimation of the prevalence and in particular of the larval burden. The predilection muscle groups vary by host species [10], and for detailed comparisons, sampling the exact same muscle within each host species would be optimal.

The background information on the animals was provided by the hunters, and the authors had no means to validate these data. Misclassification of some animals to wrong age category or sex remain possible, and no data were provided for many animals (Table 1; Table S1).

The methodology we used is harmonized at international level and thus yields comparable results. The prevalence estimates reported in this study are generally in line with results from previous studies focusing on these host species in Estonia, which estimated the prevalence to be 0.3–1.0% in wild boars, 29.4% in brown bears, and 47.4–50% in lynxes [8,18]. The proportion of badgers that tested positive in this study was significantly higher (60.0%, three of five, *p* = 0.006) than an earlier estimate for badgers hunted in 1965–2000 (6.7%, six of 89) [19].

While the results of this study are not directly comparable with those from other countries due to different sampling and study designs, it is clear that *Trichinella* parasites thrive in the region. The prevalence in wild boars in this study was lower than that observed in Latvia [20], but higher than that in Poland [21]. The prevalence in brown bears and lynxes was higher than that in Finland [22], while the prevalence in lynxes was lower than that in Latvia [8,23]. The prevalence in badgers is considered low in several countries [24], however the proportion of positives in this study was substantial, in line with what has been observed in Latvia, and higher than that in Finland [22,23].

The results of this study confirm that *T. britovi* has been winning host-terrain, while *T. nativa* is well-established in whole mainland Estonia. It is noteworthy that *T. pseudospiralis* was found in animals from the southwestern part of the country. Several studies have described an increase of *T. pseudospiralis* findings in wild boar samples in Europe [25]. One possible vector of *T. pseudospiralis* are predatory birds, including migratory birds [25]. In Estonia, the findings of *T. pseudospiralis* have been made in animals killed near the sea or wetlands areas, which are good nesting sites for birds. Further research focusing on the potential host species living in these specific environments could provide insight into the role of birds in the epidemiology of *T. pseudospiralis*. Another noteworthy finding was *T. spiralis* from a lynx killed in 2009 in Järvamaa county, approximately 30 km from where a human trichinellosis outbreak was documented ten years earlier, and where *T. spiralis* was found in a domestic pig during the investigation [18]. *Trichinella spiralis* could be infecting wildlife in Estonia similarly as described by Oksanen and others [22], as spillover from the domestic cycle. That we did not find freeze-sensitive *T. spiralis* in the main reservoir animal host species in our previous study [6] might be explained by two consecutive colder years before 2011/2012 [26].

In contrast to our previous epidemiological study in the reservoir hosts raccoon dogs and red foxes, where no obvious geographical differences in *Trichinella* prevalence were seen [6], geographical differences in the prevalence were observed in wild boars and lynxes in this study. Interestingly, we previously demonstrated a higher seroprevalence in wild boars in the southwestern part of the country [7], and the results of this study confirm a higher infection prevalence in western and southern counties. The geographical variation may be due to several factors, including climate, temperature, and snow cover [16,20].

The results of this study exemplify that wild boars can serve as an indicator for *Trichinella* spp. monitoring, being annually hunted in high numbers and routinely tested for *Trichinella*. Wild boars have been popular game in Estonia after their population rapidly increased since the second half of the 1990s, supported by relatively mild winters and supplementary feeding [27,28,29,30]. Importantly, the results of this study reflect the situation before the African swine fever (ASF) outbreak in Estonia, which started in September 2014, and will thus serve as baseline data for future studies that could evaluate how the ASF-related changes affected the wild boar population and the parasites these animals can host.

The results of this study highlight that testing wildlife hunted for human consumption for *Trichinella* remains important, and that there is room for improvement in the proportion of hunted animals tested. Wildlife are important for epidemiology of *Trichinella* spp. in Estonia, and hunting wild game for human consumption provides a potential transmission route to humans.

## 5. Conclusions

In Estonia, *Trichinella* infections are common in wildlife, including in game animals hunted for human consumption. High infection pressure was evident in sylvatic cycles, and the risk for spillover to and from domestic cycles and transmission to humans remain relevant.

## Figures and Tables

**Table 1:** Prevalence of *Trichinella* infection in wild boars (*Sus scrofa*), brown bears (*Ursus arctos*), Eurasian lynxes (*Lynx lynx*) and badgers (*Meles meles*) hunted in Estonia, 2007–2014, by sex, age category, region, and year. Univariable odds to test positive in comparison to the reference sex (male), age category (juvenile), east-west category (eastern counties), south-north category (southern counties), and year (2007) are shown for each variable, and larval burden data and the *Trichinella* species identified are summarized.

Variable	N Tested ^a^	n pos ^a^ (n pos ^a, b^)	Prevalence (95% CI) ^a^	Odds Ratio (95% CI) ^a^	*p*-Value ^a^	Median lpg ^a,b^	Mean lpg ^a,b^	Range lpg ^a,b^	*Trichinella* Species Identified (n Animals) ^a,b^
**Wild boars**									
**Sex**									
**Male**	2631	65 (66)	2.5 (1.9–3.1)	reference	−	1.43	11.89	0.02–101.08	Tb (52), Tn (6), Tp (1), Tb+Tn (2), Tb+Ts (1), Tspp (4)
**Female**	1386	45 (47)	3.2 (2.4–4.3)	1.3 (0.9–1.9)	0.157	0.58	10.59	0.02–100.00	Tb (40), Tn (2), Tb+Ts (1), Tspp (4)
**Unknown**	26,549	171 (183)	0.6 (0.6–0.7)			2.12 ^c^	16.30 ^c^	0.01–654.50 ^c^	Tb (123), Tn (7), Ts (5), Tp (5), Tb+Tn (3), Tb+Ts (1), Tspp (39)
**Age category**									
**Juvenile**	3992	67 (68)	1.7 (1.3–2.1)	reference	−	0.82	12.56	0.02–100.00	Tb (54), Tn (5), Tb+Ts (1), Tspp (8)
**Adult**	2057	60 (62)	2.9 (2.3–3.7)	1.7 (1.2–2.4)	0.003 **	1.57	9.87	0.02–101.08	Tb (53), Tn (2), Tb+Tn (2), Tb+Ts (1), Tspp (4)
**Unknown**	24,517	154 (166)	0.6 (0.5–0.7)			2.18 ^c^	16.86 ^c^	0.01–654.50 ^c^	Tb (108), Tn (8), Ts (5), Tp (6), Tb+Tn (3), Tb+Ts (1), Tspp (35)
**Region**									
**Eastern counties**	8449	76 (79)	0.9 (0.7–1.1)	reference	−	0.83	11.17	0.02–230.88	Tb (47), Tn (10), Ts (1), Tb+Tn (2), Tspp (19)
**Western counties**	10,054	196 (208)	1.95 (1.7–2.2)	2.2 (1.7–2.9)	<0.001 ***	2.44	15.99	0.01–654.50	Tb (162), Tn (5), Tp (6), Ts (4) Tb+Tn (3), Tb+Ts (3), Tspp (25)
**Southern counties**	8204	103 (115)	1.3 (1.0–1.5)	reference	−	2.58	12.95	0.01–190.00	Tb (75), Tn (7), Tp (6), Ts (3), Tb+Tn (4), Tspp (20)
**Northern counties**	10,299	169 (172)	1.6 (1.4–1.9)	1.3 (1.0–1.7)	0.026 *	1.43	15.89	0.02–654.50	Tb (134), Tn (8), Ts (2), Tb+Tn (1), Tb+Ts (3), Tspp (24)
**Unknown**	12,063	9 (9)	0.1 (0.04–0.1)			1.73 ^c^	3.25 ^c^	0.06–12.54 ^c^	Tb (6), Tspp (3)
**Year**									
**2007**	2422	10 (12)	0.4 (0.2–0.7)	reference	−	2.55	26.15	0.10–100.00	Tb (4), Tspp (8)
**2008**	2758	10 (13)	0.4 (0.2–0.6)	0.9 (0.4–2.2)	0.774	6.00 ^c^	13.01 ^c^	0.42–58.00 ^c^	Tb (3), Tspp (10)
**2009**	4380	30 (37)	0.7 (0.5–1.0)	1.7 (0.8–3.6)	0.160	2.40	8.92	0.01–52.15	Tb (20), Tp (2), Ts (5), Tb+Ts (1), Tspp (9)
**2010**	3598	26 (27)	0.7 (0.5–1.0)	1.8 (0.9–3.8)	0.127	2.68	13.72	0.02–100.00	Tb (22), Tn (1), Tb+Tn (1), Tspp (3)
**2011**	2713	33 (35)	1.2 (0.9–1.7)	3.0 (1.5–6.3)	0.001 ***	3.44	17.88	0.02–230.88	Tb (25), Tn (3), Tb+Tn (2), Tspp (5)
**2012**	3986	26 (26)	0.7 (0.4–0.9)	1.6 (0.8–3.4)	0.217	0.89	31.40	0.02–654.50	Tb (20), Tn (1), Tp (2), Tspp (3)
**2013**	4715	77 (77)	1.6 (1.3–2.0)	4.0 (2.1–8.2)	<0.001 ***	1.60	9.07	0.02–60.00	Tb (66), Tn (4), Tp (1), Tb+Tn (2), Tspp (4)
**2014**	5994	69 (69)	1.2 (0.9–1.4)	2.8 (1.5–5.8)	<0.001 ***	0.82	13.59	0.02–191.36	Tb (55), Tn (6), Tp (1), Tb+Ts (2), Tspp (5)
**Wild boars total**	30,566	281 (296)	0.9 (0.8–1.0)			1.64 ^c^	14.40 ^c^	0.01–654.50 ^c^	Tb (215), Tn (15), Tp (6), Ts (5), Tb+Tn (5), Tb+Ts (3), Tspp (47)
**Brown bears**									
**Sex**									
**Male**	26	13 (13)	50.0 (31.3–68.7)	reference	-	0.20	1.17	0.02–10.96	Tb (7), Tn (1), Tspp (5)
**Female**	14	9 (9)	64.3 (37.6–85.6)	1.8 (0.5–7.3)	0.413	3.48	4.81	0.02–11.02	Tb (4), Tn (4), Tb+Tn (1)
**Unknown**	389	41 (42)	10.5 (7.8–13.9)			0.54	4.78	0.02–81.96	Tb (18), Tn (17), Tb+Tn (2), Tspp (5)
**Age category**									
**Juvenile**	22	2 (2)	9.1 (1.55–26.9)	reference	-	4.53	4.53	0.70–8.36	Tb (1), Tn (1)
**Adult**	87	19 (19)	21.8 (14.1–31.4)	2.8 (0.7–19.0)	0.185	0.86	3.00	0.02–11.02	Tb (10), Tn (5), Tb+Tn (2), Tspp (2)
**Unknown**	320	42 (43)	13.1 (9.75–17.2)			0.24	4.50	0.02–81.96	Tb (18), Tn (16), Tb+Tn (1), Tspp (8)
**Region**									
**Eastern counties**	304	48 (48)	15.8 (12.0–20.2)	reference	−	0.42	4.78	0.03–81.96	Tb (20), Tn (18), Tspp (10)
**Western counties**	72	12 (13)	16.7 (9.4–26.6)	1.1 (0.5–2.1)	0.839	0.13	2.06	0.02–10.76	Tb (7), Tn (3), Tb+Tn (3)
**Southern counties**	141	16 (17)	11.35 (6.9–17.4)	reference	−	0.14	6.27	0.02–81.96	Tb (10), Tn (4), Tb+Tn (1), Tspp (2)
**Northern counties**	235	44 (44)	18.7 (14.1–24.1)	1.8 (1.0–3.4)	0.058	0.77	3.40	0.02–29.96	Tb (17), Tn (17), Tb+Tn (2), Tspp (2)
**Unknown**	53	3 (3)	5.7 (1.5–14.6)			0.76	1.11	0.20–2.36	Tb (2), Tn (1)
**Year**									
**2007**	46	8 (8)	17.4 (8.4–30.4)	reference	−	0.35	0.95	0.03–4.10	Tb (2), Tn (1), Tspp (5)
**2008**	50	5 (5)	10.0 (3.8–20.8)	0.5 (0.1–1.8)	0.311	0.60	1.01	0.06–2.03	Tb (3), Tspp (2)
**2009**	51	7 (8)	13.7 (6.2–25.3)	0.8 (0.2–2.3)	0.631	0.44	4.71	0.06–15.90	Tb (3), Tn (3), Tspp (2)
**2010**	64	9 (9)	14.1 (7.1–24.2)	0.8 (0.3–2.3)	0.641	0.20	1.53	0.02–9.48	Tb (7), Tn (2)
**2011**	64	8 (8)	12.5 (6.0–22.4)	0.7 (0.2–2.0)	0.487	3.44	5.53	0.04–16.34	Tb (5), Tn (3)
**2012**	74	8 (8)	12.2 (6.1–21.1)	0.6 (0.2–1.7)	0.320	0.85	4.61	0.02–28.96	Tb (1), Tn (5), Tb+Tn (2)
**2013**	42	15 (15)	35.7 (22.4–51.0)	2.6 (1.0–7.4)	0.057	0.19	2.43	0.06–11.02	Tb (7), Tn (6), Tb+Tn (1), Tspp (1)
**2014**	38	3 (3)	7.9 (2.1–20.0)	0.4 (0.1–1.6)	0.220	0.10	27.39	0.10–81.96	Tb (1), Tn (2)
**Brown bears total**	429	63 (64)	14.7 (11.6–18.3)			0.44	4.11	0.02–81.96	Tb (29), Tn (22), Tb+Tn (3), Tspp (10)
**Lynxes**									
**Sex**									
**Male**	14	14 (14)	100.0 (80.7–100.0)	reference	−	2.38	6.51	0.20–28.00	Tb (9), Tn (1), Tb+Tn (4)
**Female**	10	8 (8)	80.0 (48.1–96.5)	−	0.163	0.90	4.14	0.38–20.10	Tb (3), Tn (2), Ts (1), Tb+Tn (1), Tspp (1)
**Unknown**	66	37 (41)	56.1 (44.0–67.7)			1.20 ^c^	3.53 ^c^	0.02–21.40 ^c^	Tb (15), Tn (1), Ts (3), Tb+Tn (10), Tb+Ts (1), Tspp (12)
**Age category**									
**Juvenile**	15	7 (7)	46.7 (23.2–71.3)	reference	−	14.88	10.97	0.20–28.00	Tb (3), Ts (1), Tb+Tn (3)
**Adult**	17	14 (14)	82.4 (59.1–95.3)	5.0 (1.0–30.2)	0.045 *	1.91	2.78	0.04–9.22	Tb (6), Tn (2), Ts (1), Tb+Tn (4), Tspp (1)
**Unknown**	58	38 (42)	65.5 (52.7–76.9)			0.99 ^c^	3.65 ^c^	0.02–21.40 ^c^	Tb (18), Tn (2), Ts (2), Tb+Tn (8), Tb+Ts (1), Tspp (1)
**Region**									
**Eastern counties**	60	36 (38)	60.0 (47.3–71.8)	reference	−	1.19 ^c^	2.76 ^c^	0.02–21.40 ^c^	Tb (16), Tn (1), Ts (1), Tb+Tn (8), Tb+Ts (1), Tspp (11)
**Western counties**	27	23 (25)	85.2 (68.0–95.1)	3.8 (1.2–14.2)	0.020 *	2.34	6.49	0.1–28.00	Tb (11), Tn (3), Ts (3), Tb+Tn (7), Tspp (1)
**Southern counties**	33	18 (21)	54.5 (37.5–70.8)	reference	−	2.42	3.66	0.02–14.60	Tb (9), Tn (1), Ts (2), Tb+Tn (9)
**Northern counties**	54	41 (42)	75.9 (63.2–85.9)	2.6 (1.0–6.7)	0.045 *	1.14 ^c^	4.62 ^c^	0.16–28.00 ^c^	Tb (18), Tn (3), Ts (2), Tb+Tn (6), Tb+Ts (1), Tspp (12)
**Unknown**	3	0 (0)	0.0 (0.0–63.2)			−	−	−	−
**Year**									
**2007**	10	5 (6)	50.0 (21.2–78.8)	reference	−	1.45 ^c^	6.25 ^c^	0.70–21.40 ^c^	Tb+Tn (1), Tspp (5)
**2008**	12	5 (5)	41.7 (17.2–69.8)	0.7 (0.1–4.2)	0.721	0.90	2.18	0.50–7.60	Tb (3), Tspp (2)
**2009**	20	14 (16)	70.0 (47.7–86.8)	2.3 (0.5–11.8)	0.321	0.50	2.08	0.10–14.88	Tb (5), Ts (4), Tb+Tn (3), Tb+Ts (1), Tspp (3)
**2010**	17	11 (12)	64.7 (40.5–84.3)	1.8 (0.35–9.5)	0.487	0.82	2.73	0.20–14.60	Tb (9), Tn (1), Tb+Tn (2)
**2011**	11	7 (7)	63.6 (33.6–87.2)	1.7 (0.3–10.9)	0.567	2.48	2.48	0.02–4.82	Tb (4), Tn (1), Tb+Tn (2)
**2012**	11	10 (10)	90.9 (62.7–99.6)	8.9 (0.9–259.1)	0.059	8.39	10.10	0.04–28.00	Tb (4), Tn (1), Tb+Tn (3), Tspp (2)
**2013**	9	7 (7)	77.8 (43.8–96.1)	3.3 (0.4–33.1)	0.260	6.24	6.23	0.38–12.10	Tb (2), Tn (1), Tb+Tn (4)
**Lynxes total**	90	59 (63)	65.6 (55.3–74.8)			1.42 ^c^	4.29 ^c^	0.02–28.00 ^c^	Tb (27), Tn (4), Ts (4), Tb+Tn (15), Tb+Ts (1), Tspp (12)
**Badgers**									
**Sex**									
**Male**	1	1	100.0 (5.0–100.0)	−	−	20.96	20.96	20.96	Tb (1)
**Female**	1	0	0.0 (0.0–95.0)	−	−	−	−	−	−
**Unknown**	3	2	66.7 (13.2–98.3)			7.09	7.09	2.90–11.28	Tb (1), Tspp (1)
**Region**									
**Eastern counties**	3	2	66.7 (13.2–98.3)	−	−	10.09	10.09	10.09	Tb (1), Tspp (1)
**Western counties**	2	1	50.0 (2.5–97.5)	−	−	20.96	20.96	20.96	Tb (1)
**Southern counties**	3	2	66.7 (13.2–98.3)	−	−	11.93	11.93	11.93	Tb (1), Tspp (1)
**Northern counties**	2	1	50.0 (2.5–97.5)	−	−	11.28	11.28	11.28	Tb (1)
**Year**									
**2008**	1	1	100.0 (5.0–100.0)	−	−	2.90	2.90	2.90	Tb (1)
**2013**	2	2	100.0 (22.4–100.0)	−	−	16.12	16.12	11.28–20.96	Tb (1), Tspp (1)
**2014**	2	0	0.0 (0.0–77.6)	−	−	−	−	−	−
**Badgers total**	5	3 (3)	60.0 (18.2–92.7)			11.28	11.71	2.90–20.96	Tb (2), Tspp (1)

N Tested: total number of animals tested; ^a^ Animals tested as primary testing; ^b^ Positive animals tested for confirmatory purposes.; ^c^ No data on larval burden for one wild boar and two lynxes; 95% CI: 95% confidence interval, Mid-P Exact; n pos: number of *Trichinella* positive animals; lpg: number of *Trichinella* larvae per gram of muscle tissue; n Animals: number of animals; *p*-value: two-tailed Mid-P Exact: *: *p* ≤ 0.05; **: *p* ≤ 0.01; ***: *p* ≤ 0.001; Eastern counties: Ida-Virumaa, Jõgevamaa, Järvamaa, Lääne-Virumaa, Põlvamaa, Tartumaa, Valgamaa; Võrumaa; Western counties: Harjumaa, Hiiumaa, Läänemaa, Pärnumaa, Raplamaa, Saaremaa, Viljandimaa; Southern counties: Jõgevamaa, Põlvamaa, Pärnumaa, Saaremaa, Tartumaa, Valgamaa, Viljandimaa, Võrumaa; Northern counties: Harjumaa, Hiiumaa, Ida-Virumaa, Järvamaa, Läänemaa, Lääne-Virumaa, Raplamaa; Tb: *Trichinella britovi*; Tn: *Trichinella nativa*; Tp: *Trichinella pseudospiralis;* Ts: *Trichinella spiralis*; Tb+Tn: mixed infection with *Trichinella britovi* and *Trichinella nativa*; Tb+Ts: mixed infection with *Trichinella britovi* and *Trichinella spiralis;* Tspp: *Trichinella* species, no species-level result.

**Table 2:** *Trichinella* species identified in wild boars (*Sus scrofa*), brown bears (*Ursus arctos*), Eurasian lynxes (*Lynx lynx*) and badgers (*Meles meles*) hunted in 2007–2014 in Estonia.

*Trichinella* Species	Wild Boars (n = 30,566 ^a^+15 ^b^)				Brown Bears (n = 429 ^a^ +1 ^b^)				Lynxes (n = 90 ^a^ +4 ^b^)				Badgers (n = 5 ^a^)			
	n pos ^a^ (n pos ^a,b^)	Prevalence % (95% CI) ^a^	% (95% CI) of *Trichinella* Positive ^a,b^	Median; Mean (Range) of lpg ^a,b^	n pos ^a^ (n pos ^a,b^)	Prevalence % (95% CI) ^a^	% (95% CI) of *Trichinella* Positive ^a,b^	Median; Mean (Range) of lpg ^a,b^	n pos ^a^ (n pos ^a,b^)	Prevalence % (95% CI) ^a^	% (95% CI) of *Trichinella* Positive ^a,b^	Median; Mean (Range) of lpg ^a,b^	n pos^a^ (n pos ^a,b^)	Prevalence % (95% CI) ^a^	% (95% CI) of *Trichinella* Positive ^a,b^	Median; Mean (Range) of lpg ^a,b^
*T. britovi* only	209 (215)	0.7 (0.6–0.8)	72.6 (67.3–77.5)	2.06; 16.30 (0.02–654.5)	28 (29)	6.5 (4.5–9.2)	45.3 (33.5–57.6)	0.48; 5.88 (0.10–81.96)	26 (27)	28.9 (20.2–38.9)	42.9 (31.1–55.3)	1.48; 4.71 (0.06–28.00)	2 (2)	40.0 (7.4–81.8)	66.7 (13.2–98.3)	16.12; 16.12 (11.28–20.96)
*T. nativa* only	15 (15)	0.05 (0.03–0.08)	5.1 (3.0–8.0)	0.82; 6.69 (0.04–54.32)	22 (22)	5.1 (3.3–7.5)	34.4 (23.5–46.6)	0.60; 3.40 (0.02–28.96)	4 (4)	4.4 (1.4–10.4)	6.3 (2.1–14.6)	3.53; 6.80 (0.02–20.10)	−	−	−	−
*T. pseudospiralis* only	6 (6)	0.02 (0.01–0.04)	2.0 (0.8–4.2)	1.63; 5.52 (0.18–23.34)	−	−	−	−	−	−	−	−	−	−	−	−
*T. spiralis* only	5 (5)	0.02 (0.01–0.04)	1.7 (0.6–3.7)	4.76; 4.51 (0.20–7.65)	−	−	−	−	3 (4)	3.3 (0.9–8.8)	6.3 (2.1–14.6)	3.61; 5.58 (0.23–14.88)	−	−	−	−
*T. britovi* and *T. nativa*	5 (5)	0.02 (0.01–0.04)	1.7 (0.6–3.7)	1.38; 10.58 (0.04–45.20)	3 (3)	0.7 (0.2–1.9)	4.7 (1.2–12.2)	3.48; 2.41 (0.02–3.74)	14 (15)	15.6 (9.1–24.2)	23.8 (14.5–35.5)	2.00; 4.62 (0.04–17.40)	−	−	−	−
*T. britovi* and *T. spiralis*	3 (3)	0.01 (0.00–0.03)	1.0 (0.3–2.7)	0.24; 2.67 (0.16–7.60)	−	−	−	−	1 (1)	1.1 (0.1–5.4)	1.6 (0.1–7.6)	0.20	−	−	−	−
*T. britovi* Total ^c^	217 (223)	0.7 (0.6–0.8)	75.3 (70.2–80.0)	1.88; 15.99 (0.02–654.50)	31 (32)	7.2 (5.1–10.0)	50.0 (37.9–51.4)	0.54; 5.56 (0.02–81.96)	41 (43)	45.6 (35.5–55.9)	68.3 (56.0–78.8)	1.58; 4.23 (0.04–28.00)	2 (2)	40.0 (7.4–81.8)	66.7 (13.2–98.3)	16.12; 16.12 (11.28–20.96)
*T. nativa* Total ^c^	20 (20)	0.07 (0.04–0.10)	6.8 (4.3–10.1)	1.14; 7.66 (0.04–54.32)	25 (25)	5.8 (3.9–8.4)	39.1 (27.7–51.4)	0.76; 3.28 (0.02–28.96)	18 (19)	20.0 (12.7–29.2)	30.2 (19.8–42.3)	2.00; 5.08 (0.02–20.10)		−	−	−
*T. pseudospiralis* total ^c^	6 (6)	0.02 (0.01–0.04)	2.0 (0.8–4.2)	3.70; 6.21 (0.18–23.34)	−	−	−	−	−	−	−	−		−	−	−
*T. spiralis* Total ^c^	8 (8)	0.03 (0.01–0.05)	2.7 (1.3–5.1)	4.26; 3.82 (0.16–7.65)	−	−	−	−	4 (5)	4.4 (1.4–10.4)	7.9 (3.0–16.7)	3.60; 4.51 (0.20–14.88)		−	−	−
Species-level result total	243 (249)	0.8 (0.7–0.9)	84.1 (79.6–88.0)	1.76; 15.07 (0.02–654.50)	53 (54)	12.4 (9.5–15.7)	84.4 (73.9–91.8)	0.60; 4.76 (0.02–81.96)	48 (51)	53.3 (43.0–63.5)	81.0 (69.9–89.3)	1.64; 4.54 (0.02–28.00)	2 (2)	40.0 (7.4–81.8)	66.7 (13.2–98.3)	16.12; 16.12 (11.28–20.96)
No species-level result	38 (47)	0.1 (0.1–0.2)	15.9 (12.0–20.4)	0.70; 10.55 (0.01–190.00) ^d^	10 (10)	2.3 (1.2–4.1)	15.6 (8.2–26.1)	0.23; 0.68 (0.03–2.03)	11 (12)	12.2 (6.6–20.3)	19.0 (10.8–30.1)	0.65; 3.02 (0.16–21.40) ^d^	1 (1)	20.0 (1.0–66.6)	33.3 (1.7–86.8)	2.9
Total	281 (296)	0.9 (0.8–1.0)	100.0	1.64; 14.40 (0.01–654.50) ^d^	63 (64)	14.7 (11.6–18.3)	100.0	0.42; 4.05 (0.02–81.96)	59 (63)	65.6 (55.3–74.8)	100.0	1.45; 4.46 (0.02–28.00) ^d^	3 (3)	60.0 (18.2–92.7)	100.0	11.28; 11.71 (290–20.96)

^a^ Animals tested as primary testing; ^b^ Positive animals tested for confirmatory purposes; ^c^ The number of animals with the *Trichinella* species, either as the only species (mono-species infection) or in mixed infection; ^d^ No data on larval burden for one wild boar and two lynxes; 95% CI: 95% confidence interval, Mid-P Exact; n pos: number of *Trichinella* positive animals; lpg: number of *Trichinella* larvae per gram of muscle tissue.

## Data Availability

Data is contained within the article or supplementary material.

## Supplementary Materials

The following are available online at https://www.mdpi.com/2076-2615/11/1/183/s1: Table S1: Primary testing included in the study: number of wild boars (*Sus scrofa*), brown bears (*Ursus arctos*), Eurasian lynxes (*Lynx lynx*) and badgers (*Meles meles*) tested for *Trichinella* in Estonia, 2007–2014, by sex, age category, county, and by year. Table S2: Prevalence of *Trichinella* infection in wild boars (*Sus scrofa*), brown bears (*Ursus arctos*), Eurasian lynxes (*Lynx lynx*) and badgers (*Meles meles*) hunted in Estonia, 2007–2014, by county. Univariable odds to test positive in comparison to the reference county (Harjumaa), and larval burden data and the *Trichinella* species identified are summarized.
