# Supplementary material for: Trichinella spp. in Wild Boars (Sus scrofa), Brown Bears (Ursus arctos), Eurasian Lynxes (Lynx lynx) and Badgers (Meles meles) in Estonia, 2007–2014
Source: Animals (Basel). 2021 Jan 14;11(1):183. doi: 10.3390/ani11010183 (PMC7830479; doi:10.3390/ani11010183)
Supplement: Supplementary file 1 [file animals-11-00183-s001.pdf]

Table 1. Primary testing included in the study: number of wild boars (Sus scrofa), brown bears (*Ursus arctos*), Eurasian lynxes (*Lynx lynx*) and badgers (*Meles meles*) tested for *Trichinella* in Estonia, 2007–2014, by sex, age category, county, and by year.

	**Total**	**2007**	**2008**	**2009**	**2010**	**2011**	**2012**	**2013**	**2014**
Wild boars									
Sex									
Male	2631	4	0	1	8	8	88	1087	1435
Female	1386	1	2	0	4	5	55	561	758
Unknown	26,549	2417	2756	4379	3586	2700	3843	3067	3801
Age category									
Juvenile	3992	170	163	204	171	165	399	1221	1499
Adult	2057	63	64	102	92	73	140	634	889
Unknown	24,517	2189	2531	4074	3335	2475	3447	2860	3606
County									
Harjumaa	2554	125	165	178	134	98	495	565	794
Hiiumaa	304	5	1	6	0	3	92	87	110
Ida-Virumaa	473	22	44	22	45	35	99	80	126
Jõgevamaa	1078	12	20	53	45	81	185	246	436
Järvamaa	1038	26	22	37	22	25	200	308	398
Läänemaa	1995	52	32	69	64	74	478	578	648
Lääne-Virumaa	2698	222	206	235	185	182	385	463	820
Põlvamaa	1349	57	92	118	134	137	299	328	184
Pärnumaa	1156	12	11	23	18	15	326	384	367
Raplamaa	1237	50	44	68	35	30	242	291	477
Saaremaa	2438	74	27	18	37	38	532	719	347
Tartumaa	1060	40	57	64	58	79	172	243	993
Valgamaa	256	9	8	20	10	19	41	94	55
Viljandimaa	370	2	10	35	42	7	79	89	106
Võrumaa	497	7	28	16	58	59	90	147	92
Unknown	12,063	1707	1991	3418	2711	1831	271	93	41
Total	30,566	2422	2758	4380	3598	2713	3986	4715	5994
Brown bears									
Sex									
Male	26	4	2	1	2	1	3	10	3
Female	14	0	0	2	2	1	1	7	1
Unknown	389	42	48	48	60	62	70	25	34
Age category									
Juvenile	22	1	0	1	7	9	4	0	0
Adult	87	4	3	6	18	22	14	17	3

Unknown	320	41	50	51	64	64	74	42	38
County									
Harjumaa	31	0	2	4	5	3	10	3	4
Ida-Virumaa	72	14	10	13	4	5	9	8	9
Jõgevamaa	61	5	5	4	7	12	13	6	9
Järvamaa	37	2	3	4	3	8	8	6	3
Läänemaa	2	0	0	1	1	0	0	0	0
Lääne-Virumaa	75	5	8	9	12	15	15	6	5
Põlvamaa	14	2	5	0	0	2	2	2	1
Pärnumaa	14	0	2	3	4	2	1	0	2
Raplamaa	18	1	3	1	3	3	5	1	1
Tartumaa	44	3	6	7	5	3	9	8	3
Viljandimaa	7	0	2	1	1	0	1	1	1
Võrumaa	1		1						
Unknown	53	14	3	4	19	11	1	1	0
Total	429	46	50	51	64	64	74	42	38
Lynxes									
Sex									
Male	14	0	2	1	4	0	4	3	0
Female	10	0	1	1	4	0	1	3	0
Unknown	66	10	9	18	9	11	6	3	0
Age category									
Juvenile	15	0	1	1	5	2	3	3	0
Adult	17	1	1	2	5	2	3	3	0
Unknown	58	9	10	17	7	7	5	3	0
County									
Harjumaa	9	0	1	2	3	1	0	2	0
Ida-Virumaa	9	1	6	1	1	0	0	0	0
Jõgevamaa	10	0	0	1	4	3	0	2	0
Järvamaa	1	0	0	1	0	0	0	0	0
Läänemaa	7	1	0	0	3	1	1	1	0
Lääne-Virumaa	23	4	3	10	1	2	3	0	0
Põlvamaa	10	0	2	2	1	3	1	1	0
Pärnumaa	2	0	0	1	1	0	0	0	0
Raplamaa	5	0	0	0	0	0	5	0	0
Tartumaa	4	0	0	0	0	1	1	2	0
Valgamaa	1	1	0	0	0	0	0	0	0
Viljandimaa	4	0	0	1	2	0	0	1	0
Võrumaa	2	0	0	1	1	0	0	0	0
Unknown	3	3	0	0	0	0	0	0	0
Total	90	10	12	20	17	11	11	9	0

Badgers									
Sex									
Male	1	0	0	0	0	0	0	1	0
Female	1	0	0	0	0	0	0	0	1
Unknown	3	0	1	0	0	0	0	1	1
County									
Jõgevamaa	1	0	1	0	0	0	0	0	0
Järvamaa	1	0	0	0	0	0	0	0	1
Lääne-Virumaa	1	0	0	0	0	0	0	1	0
Pärnumaa	1	0	0	0	0	0	0	0	1
Viljandimaa	1	0	0	0	0	0	0	1	0
Total	5	0	1	0	0	0	0	2	2
Animals total	31,090	2478	2821	4451	3679	2788	4071	4768	6034

Table 2. Prevalence of *Trichinella* infection in wild boars (*Sus scrofa*), brown bears (*Ursus arctos*), Eurasian lynxes (*Lynx lynx*) and badgers (*Meles meles*) hunted in Estonia, 2007–2014, by county. Univariable odds to test positive in comparison to the reference county (Harjumaa), and larval burden data and the *Trichinella* species identified are summarized.

County	**N tested** [a]	**n pos** [a] **(n pos** [a, b]**)**	**Prevalence (95% CI)** [a]	**Odds ratio (95% CI)** [a]	***p*-value** [a]	**Median lpg** [a, b]	**Mean lpg** [a, b]	**Range lpg** [a, b]	***Trichinella* species identified (n animals)** [a, b]
Wild boars									
Harjumaa	2554	51 (52)	2.0 (1.5–2.6)	reference	-	1.64	23.45	0.02–654.50	Tb (44), Tn (2), Tb+Tn (1), Tb+Ts (2), Tspp (3)
Hiiumaa	304	8 (8)	2.6 (1.2–4.9)	1.3 (0.6–2.7)	0.454	3.60	14.68	0.02–55.50	Tb (7), Tspp (1)
Ida-Virumaa	473	9 (11)	1.9 (0.9–3.5)	0.95 (0.4–1.9)	0.924	1.90	12.61	0.02–100.00	Tb (4), Tn (2), Tspp (5)
Jõgevamaa	1078	12 (12)	1.1 (0.6–1.9)	0.55 (0.3–1.0)	0.057	0.59	5.21	0.02–45.20	Tb (4), Tn (4), Tb+Tn (1), Tspp (3)
Järvamaa	1038	13 (13)	1.3 (0.7–2.1)	0.6 (0.3–1.1)	0.123	1.88	13.88	0.02–101.08	Tb (10), Tn (1), Tspp (2)
Läänemaa	1995	42 (42)	2.1 (1.5–2.8)	1.1 (0.7–1.6)	0.796	0.83	10.10	0.02–191.36	Tb (37), Tn (1), Ts (1), Tspp (3)
Lääne-Virumaa	2698	25 (25)	0.9 (0.6–1.3)	0.5 (0.3–0.7)	0.001 ***	0.76	14.32	0.02–230.88	Tb (18), Tn (2), Tspp (5)
Põlvamaa	1349	4 (4)	0.3 (0.1–0.7)	0.1 (0.04–0.4)	< 0.001 ***	7.80	8.22	0.20–17.06	Tb (2), Ts (1), Tspp (1)
Pärnumaa	1156	27 (30)	2.3 (1.6–3.3)	1.2 (0.7–1.9)	0.504	1.34	8.62	0.01–100.00	Tb (23), Tn (2), Tp (1), Tb+Tn (2), Tspp (2)
Raplamaa	1237	21 (21)	1.7 (1.1–2.5)	0.8 (0.5–1.4)	0.536	7.60	12.99	0.02–53.42	Tb (14), Ts (1), Tb+Ts (1), Tspp (5)
Saaremaa	2438	45 (53)	1.8 (1.4–2.4)	0.9 (0.6–1.4)	0.700	5.43	19.06	0.04–190.00	Tb (36), Tp (5), Ts (1), Tspp (12)
Tartumaa	1060	9 (9)	0.8 (0.4–1.6)	0.4 (0.2–0.8)	0.010 **	0.14	7.24	0.02–54.32	Tb (6), Tn (1), Tb+Tn (1)
Valgamaa	256	3 (3)	1.2 (0.3–3.2)	0.6 (0.1–1.7)	0.378	0.10	0.52	0.08–1.38	Tb (2), Tspp (1)
Viljandimaa	370	2 (2)	0.5 (0.1–1.8)	0.3 (0.04–0.9)	0.034 *	5.05	5.05	4.76–5.34	Tb (1), Ts (1)
Võrumaa	497	1 (2)	0.2 (0.01–1.0)	0.1 (0.0–0.5)	0.001 ***	5.68	5.68	5.36–6.00	Tb (1), Tspp (1)
Unknown	12,063	9 (9)	0.1 (0.04–0.1)			1.73 [c]	3.25 [c]	0.06–12.54 [c]	Tb (6), Tspp (3)
Brown bears									
Harjumaa	31	7 (7)	22.6 (10.6–39.6)	reference	-	0.86	2.17	0.02–7.00	Tb (3), Tn (2), Tb+Tn (2)
Ida-Virumaa	72	12 (12)	16.7 (9.4–26.6)	0.7 (0.2–2.1)	0.488	0.78	3.15	0.03–16.34	Tb (4), Tn (2), Tspp (6)

Jõgevamaa	61	6 (6)	9.8 (4.1–19.3)	0.4 (0.1–1.3)	0.118	0.17	1.22	0.04–6.00	Tb (4), Tn (2)
Järvamaa	37	10 (10)	27.0 (14.6–42.9)	1.3 (0.4–4.0)	0.688	0.46	1.65	0.06–8.36	Tb (4), Tn (5), Tspp (1)
Läänemaa	2	2 (2)	100.0 (22.4–100.0)	3.3 (0.3–36.5)	0.312	5.39	5.39	0.02–10.76	Tb (2)
Lääne-Virumaa	75	13 (13)	17.3 (10.0–27.2)	0.7 (0.3–2.1)	0.536	0.84	5.32	0.08–28.96	Tb (4), Tn (8), Tspp (1)
Põlvamaa	14	1 (1)	7.1 (0.4–30.5)	0.3 (0.0–1.9)	0.212	0.14	0.14	0.14	Tb (1)
Pärnumaa	14	2 (2)	14.3 (2.5–39.7)	0.5 (0.1–2.6)	0.446	0.31	0.31	0.02–0.60	Tb (1), Tb+Tn (1)
Raplamaa	18	0 (0)	0.0 (0.0–15.3)	0.0 (0.0–0.8)	0.031*	-	-	-	-
Tartumaa	44	6 (6)	13.6 (5.7–26.2)	0.5 (0.15–1.9)	0.336	0.16	16.39	0.06–81.96	Tb (3), Tn (1), Tspp (2)
Viljandimaa	7	0 (1)	0.0 (0.0–34.8)	0.0 (0.0–2.3)	0.208	0.07	0.07	0.02–0.13	Tb (1), Tn (1)
Võrumaa	1	0 (0)	0.0 (0.0–95.0)	0.0 (0.0–67.9)	0.781	-	-	-	-
Unknown	53	3 (3)	5.7 (1.5–14.6)			0.76	1.11	0.2–2.36	Tb (2), Tn (1)
Lynxes									
Harjumaa	9	7 (7)	77.8 (43.8–96.1)	reference	-	0.82	4.34	0.38–14.88	Tb (3), Tn (1), Ts (1), Tb+Tn (2)
Ida-Virumaa	9	6 (6)	66.7 (33.2–90.7)	0.6 (0.1–5.3)	0.647	0.60[c]	0.68[c]	0.24–1.30[c]	Tb (4), Tspp (2)
Jõgevamaa	10	5 (5)	50.0 (21.2–78.8)	0.3 (0.0–2.3)	0.260	2.48	2.44	0.02–4.26	Tb (2), Tn (1), Tb+Tn (2)
Järvamaa	1	1 (2)	100.0 (5.0–100.0)	-	0.800	3.73	3.73	3.60–3.86	Ts (1), Tspp (1)
Läänemaa	7	5 (5)	71.4 (33.0–94.9)	0.7 (0.1–9.1)	0.800	4.82	3.65	0.20–6.24	Tb (3), Tn (1), Tspp (1)
Lääne-Virumaa	23	17 (17)	73.9 (53.4–88.7)	0.8 (0.1–5.0)	0.858	0.84[c]	2.66[c]	0.16–21.40[c]	Tb (6), Tn (1), Tb+Tn (2), Tb+Ts (1), Tspp (8)
Põlvamaa	10	4 (4)	40.0 (14.2–70.9)	0.2 (0.0–1.5)	0.130	1.24	3.13	0.04–10.00	Tb (1), Tb+Tn (3)
Pärnumaa	2	2 (4)	100.0 (22.4–100.0)	-	0.655	0.41	0.73	0.10–2.00	Tb (1), Ts (1), Tb+Tn (2)
Raplamaa	5	5 (5)	100.0 (54.9–100.0)	-	0.396	17.4	16.55	2.34–28.00	Tb (2), Tn (1), Tb+Tn (2)

Tartumaa	4	2 (2)	50.0 (9.4–90.6)	0.3 (0.0–4.7)	0.406	8.94	8.94	4.72–13.16	Tb (1), Tb+Tn (1)
Valgamaa	1	0 (1)	0.0 (0.0–95.0)	0.0 (0.0–8.1)	0.300	0.54	0.54	0.54	Tb (1)
Viljandimaa	4	4 (4)	100.0 (47.3–100.0)	–	0.462	6.42	6.98	0.46–14.60	Tb (2), Ts (1), Tb+Tn (1)
Võrumaa	2	1 (1)	50.0 (2.5–97.5)	0.3 (0.0–16.5)	0.546	2.96	2.96	2.96	Tb (1)
Unknown	3	0 (0)	0.0 (0.0–63.2)			–	–	–	–
Badgers									
Jõgevamaa	1	1	100.0 (5.0–100.0)	–	–	2.90	2.90	2.90	Tspp (1)
Järvamaa	1	0	0.0 (0.0–95.0)	–	–	–	–	–	–
Lääne-Virumaa	1	1	100.0 (5.0–100.0)	–	–	11.28	11.28	11.28	Tb (1)
Pärnumaa	1	0	0.0 (0.0–95.0)	–	–	–	–	–	–
Viljandimaa	1	1	100.0 (5.0–100.0)	–	–	20.96	20.96	20.96	Tb (1)

[a] Animals tested as primary testing; [b] Positive animals tested for confirmatory purposes.; [c] No data on larval burden for one wild boar and two lynxes. 95% CI: 95% confidence interval, Mid-P Exact; n pos: number of *Trichinella* positive animals; lpg: number of *Trichinella* larvae per gram of muscle tissue; *p*-value: two-tailed Mid-P Exact: * $p \leq 0.05$; ** $p \leq 0.01$; *** $p \leq 0.001$; Tb: *Trichinella britovi*; Tn: *Trichinella nativa*; Tp: *Trichinella pseudospiralis;* Ts: *Trichinella spiralis*; Tb+Tn: mixed infection with *Trichinella britovi* and *Trichinella nativa*; Tb+Ts: mixed infection with *Trichinella britovi* and *Trichinella spiralis;* Tspp: *Trichinella* species, no species-level result.
